# Chronic Pelvic Pain in Endometriosis: Cross-Sectional Associations with Mental Disorders, Sexual Dysfunctions and Childhood Maltreatment

**DOI:** 10.3390/jcm11133714

**Published:** 2022-06-27

**Authors:** Johanna Netzl, Burkhard Gusy, Barbara Voigt, Jalid Sehouli, Sylvia Mechsner

**Affiliations:** 1 Department of Psychology, Freie Universität Berlin, Habelschwerdter Allee 45, 14195 Berlin, Germany; johanna.netzl@charite.de (J.N.); burkhard.gusy@fu-berlin.de (B.G.); 2 Endometriosis Centre Charité, Department of Gynaecology with Center for Oncological Surgery, Charité–Universitätsmedizin Berlin, Corporate Member of Freie Universität Berlin and Humboldt-Universität zu Berlin, Augustenburger Platz 1, 13353 Berlin, Germany; jalid.sehouli@charite.de; 3 Department of Psychosomatic Medicine, Center for Internal Medicine and Dermatology, Charité–Universitätsmedizin Berlin, Corporate Member of Freie Universität Berlin and Humboldt-Universität zu Berlin, Charitéplatz 1, 10117 Berlin, Germany; barbara.voigt@charite.de

**Keywords:** childhood maltreatment, chronic pelvic pain, endometriosis, mental disorder, sexual dysfunction

## Abstract

The aim of this cross-sectional study was to compare the rates of mental disorders, sexual dysfunctions and childhood maltreatment (CM) in women with endometriosis with either chronic pelvic pain (CPP) or minimal to no pelvic pain. Additionally, two models to predict a current mental disorder were tested, including pelvic-pain-related or psychosocial predictor variables. We examined 100 women with confirmed endometriosis (group CPP, *n* = 50; group NOPAIN, *n* = 50). Participants responded to a comprehensive questionnaire and the Childhood Trauma Questionnaire. The Diagnostic Interview for Mental Disorders was used to assess mental disorders according to DSM-5 and to screen for sexual dysfunctions. The mean age was 28.8 ± 5.6 (CPP)/2.7 ± 6.3 (NOPAIN). Participants with CPP had higher rates of current mental disorders (*p* = 0.019), lifetime mental disorders (*p* = 0.006) and sexual dysfunctions (*p* < 0.001), but not CM (*p* = 0.074). In two binary-logistic regression analyses, a greater need for pain relief (aOR = 4.08, *p* = 0.026) and a sexual dysfunction (aOR = 2.69, *p* = 0.031) were significant predictors for a current mental disorder. Our findings confirmed the crucial role of pelvic pain for mental and sexual well-being in endometriosis. They highlight the need for pain relief and interdisciplinary care in the treatment of endometriosis.

## 1. Introduction

Endometriosis (EM) is an estrogen-dependent chronic inflammatory gynecological disease that affects about 10% of women of reproductive age [1]. It is characterized by the ectopic proliferation of endometrium-like tissue and is associated with infertility and various forms of pelvic pain [2]. EM is the most common cause of chronic pelvic pain (CPP) in women [1]. In some cases, however, EM is present without pain symptoms [2]. Furthermore, while some patients’ symptoms improve dramatically with treatment, others suffer from persistent pain.

While there is a growing body of literature on psychopathological symptoms in women with EM [3,4], research on manifest mental disorders in this patient group is scarce [5,6,7,8]. The assessment of psychological distress at the level of symptoms (dimensional approach) or disorders (categorical approach) is methodologically different [9]. Generally, psychopathological symptoms are assessed using questionnaires, which are economic, easy to administer and evaluate and often used as screening or monitoring tools. Questionnaires, however, do not allow one to make a diagnosis of manifest mental disorders. The international gold standard in the diagnosis of mental disorders is the use of standardized or structured clinical interviews. These comprise predefined questions, which allow the evaluation of disorder-specific syndromes, as defined by diagnostic manuals, their duration and the differential diagnosis to other mental disorders. Diagnostic interviews are elaborate and require the interviewer both to be familiar with diagnostic manuals and to create an atmosphere of trust and security. Ultimately, the categorical approach to diagnosis offers the possibility to identify psychological/psychiatric treatment needs and develop disorder-specific treatment methods [9]. Two meta-analyses [10,11] provided the result that CPP is a specific risk factor for psychological distress at the symptom level in EM; however, the association between CPP and manifest mental disorders in EM has not been studied before.

EM-associated pain symptoms, in general, can affect sexual functioning and CPP specifically was associated with depressive symptoms and sexual dysfunctions in women with EM [12,13]. Generally, CPP is a complex condition influenced by physical, emotional and psychological factors [14], with some evidence suggesting that, among others, childhood abuse may be a risk factor for the development of CPP [15] and, independently, for mental disorders [16]. Repeatedly, an association between EM and abuse experiences has been reported [17,18]; however, there are also contradictory findings within research [19].

Based on the existing literature, we hypothesized that women with EM and CPP have higher rates of comorbid mental disorders, sexual dysfunctions and CM than women with EM and minimal to no pelvic pain. Additionally, we aimed at setting up and testing two prediction models for a current mental disorder, including either i) pelvic-pain-related or ii) psychosocial characteristics to identify treatment options for an improvement in mental health in EM.

## 2. Materials and Methods

This cross-sectional, interview-based study is part of a larger project on the mental health of women with EM conducted at the Endometriosis Centre of Charité—Universitätsmedizin Berlin. Data were collected between March 2020 and January 2021. Approval was obtained from the ethics committee of Charité—Universitätsmedizin Berlin (Number: EA4/248/19). Written informed consent was obtained from all individual participants included in this study.

Participants were recruited from the hospital’s database and via public advertisements on EM-related social media accounts (e.g., Facebook and Instagram). The interviews were conducted via telephone due to the worldwide COVID-19-pandemic. After the interview, women were provided with information on how to consult adequate professional psychotherapy if necessary.

The general study criteria were participants being at least 18 years old and pre-menopausal, having received the diagnosis of EM at least 12 months prior, speaking fluent German, having no malignant disease or infection and having no current pregnancy. The additional criterion for group 1 CPP was having experienced CPP for at least 6 months, defined as a minimum of 20 pelvic pain days per month regardless of any pain medication. Group 2 NOPAIN comprised women who had suffered from minimal to no pelvic pain for at least 3 months, defined as a maximum of 6 pelvic pain days per month and a maximum pelvic pain intensity of 5 on a 10-point visual analog scale VAS (0 = no pain–10 = worst pain imaginable) in the absence of pain medication. All participants had a surgically confirmed diagnosis of EM. No criterion for the extent of EM lesions (rASRM score or ENZIAN classification) was set as it does not directly correlate with pain symptoms [20]. All women who answered our advertisements and met the study criteria were included until the sample size of *n* = 50 per group was reached.

For the assessment of mental disorders, the open access German Diagnostic Interview for Mental Disorders (DIPS) [21] was performed for each participant. The DIPS is a structured clinical interview for diagnosing current and lifetime mental disorders according to the diagnostic criteria listed in the *Diagnostic and Statistical Manual of Mental Disorders*, 5th ed. (DSM-5) [22]. It has satisfactory reliability, validity and acceptance among patients [23]. Point and lifetime prevalence rates of anxiety, bipolar and related, depressive, obsessive-compulsive and related, trauma- and stressor-related, somatic symptom and related, feeding and eating disorders and substance-related and addictive disorders were assessed. Additionally, the screening questions for sexual dysfunctions were included to assess sexual desire, sexual arousal, orgasmic disorder, dyspareunia and vaginismus. If participants reported current suicidal tendencies there was an emergency procedure protocol to follow. All DIPS interviews were performed by a trained and supervised psychologist.

Sociodemographic, clinical, EM-related and psychosocial data were gathered via a comprehensive questionnaire. A two-item socioeconomic status including the variables household income and education was calculated with possible scores between 2 and 13.5 [24]. Information on the subjective tolerance of (1 = bad–3 = good) and a subjective reduction in pain symptoms from (1 = not at all–5 = very high) hormonal medication was gathered. Data about the patient’s current pelvic pain symptoms were collected. Participants were asked to report the pain intensity of dysmenorrhea, non-menstrual cyclical pelvic pain (e.g., during ovulation), non-cyclical pelvic pain (e.g., CPP or pelvic pain while being on long-term hormonal treatment), pain at sexual intercourse, dyschezia and dysuria on a 10-point VAS. Additionally, participants were asked about the treatment-goal pain intensity on the same 10-point VAS for each pain type. As a measure of the need for pain relief, discrepancy scores between the actual pain intensity and the treatment-goal pain intensity for the six pain types were calculated (intensity *minus* treatment-goal intensity). Higher scores indicated a greater need for pain relief. The 28-item short form of the Childhood Trauma Questionnaire (CTQ) [25] was used as a screening tool for maltreatment in childhood and adolescence. It comprises five 5-item scales measuring abuse and neglect (emotional, physical and sexual abuse, emotional and physical neglect) and a 3-item scale measuring the tendency to minimize CM (trivialization) answered on a 5-point Likert scale (1 = not at all–5 = very often). Possible scores for each subscale range from 5 to 25/3 to 15 with higher scores indicating more or more severe maltreatment. Prevalence rates for each of the abuse and neglect subscales were calculated according to cut-off scores. The subscale physical abuse comprised only 4 of the 5 actual items in this study, as one item was overlooked when setting up the questionnaire (“I got hit or beaten so badly that it was noticed by someone like a teacher, neighbour, or doctor”), which does not pose a problem according to the instructions of the questionnaire. The cut-off scores of this scale were adjusted accordingly. However, as the item omitted has a high item difficulty, which means that participants are generally more likely to disagree with it because of its severity, meeting the cut-off score of the subscale physical abuse was comparatively easier. Therefore, our results regarding this subscale should be interpreted with caution. Assessed in a representative German sample, the CTQ showed satisfactory reliability except for the subscale physical neglect [25]. The authors suggest interpreting this scale with caution. Internal consistency for our sample ranged from α = 0.70 (emotional abuse) to α = 0.95 (sexual abuse) with an exception for the scale physical neglect (α = 0.43). Because of this low internal consistency, the subscale physical neglect was reported descriptively and included in group comparisons but excluded from the CTQ total score and further analyses to improve the reliability of our results. Additionally, as the subscale trivialization does not measure abuse or neglect experiences it was not included in the calculation of CM rates.

### Statistical Analyses

Statistical analyses were performed using IBM SPSS Statistics for Windows, Version 26.0. Armonk, NY, USA: IBM Corp, 2019 [26]. Data were described by descriptive statistics and frequencies. Categorical data between the groups CPP and NOPAIN were compared using χ^2^- or Fisher’s exact tests. Depending on normality, continuous data were compared with independent sample t-tests or Mann–Whitney U-tests. Values of *p* < 0.05 were considered statistically significant. Effect sizes are reported as Cohen’s d and odds ratio.

Additionally, two principal component analyses (PCA) were run regarding the pain intensity scores and the discrepancy scores between the actual and the treatment-goal pain intensity as a measure of the need for pain relief. PCA can be used to reduce a set of variables to a smaller size [27] and was applied to pain scores before [28]. In the first PCA, the VAS scores of dysmenorrhea, non-menstrual cyclical pelvic pain, non-cyclical pelvic pain, pain at sexual intercourse, dyschezia and dysuria were included. In the second PCA, the discrepancy scores of the 6 pain types were included. The results of both PCAs supported a one-component solution. The component for pain intensity (new variable PC_intensity) explained 53.62% of the variance with loadings of 0.52–0.78. The component describing the need for pain relief (new variable PC_painrelief) explained 51.77% of the variance with loadings of 0.44–0.82. By establishing these components, we were able to pool information and aimed at avoiding multicollinearity and overfitting in the subsequent regression analysis. For a more detailed description of PCA statistics, see Appendix A.

To set up and test prediction models of a current mental disorder (dichotomous dependent variable), two binary logistic regression analyses with a priori determined independent predictor variables were run. The models included either (i) pelvic-pain-related independent variables: number of pelvic pain days per month, PC_intensity and PC_painrelief; or (ii) psychosocial independent variables: CM without the subscales physical neglect and trivialization, former mental disorder and any sexual dysfunction. We decided on two separate models due to the statistical requirement regarding the number of independent variables in relation to the sample size. In binary logistic regression analysis, for each independent variable, a minimum of 10 cases per category of the dependent variable is recommended [27]. The goal of these binary logistic regression models was to test associations of pelvic-pain-related and psychosocial variables with a current mental disorder in women with EM and to derive treatment implications for an improvement in mental health in EM patients from these results. Results of binary logistic regression analyses are reported by coefficients, adjusted odds ratios with a 95% confidence interval, Hosmer–Lemeshow goodness-of-fit test, Nagelkerke’s R^2^ and receiver operating characteristics curve (ROC curve) with the area under the curve (AUC).

## 3. Results

### 3.1. Demographic, Clinical and Endometriosis-Related Data

All demographic, clinical and EM-related data, split by group, can be found in Table 1. The duration of the DIPS interviews did not differ between the groups (CPP: *M* = 96.14 ± 28.33 min, NOPAIN: *M* = 94.06 ± 32.04 min; *t*(96) = 0.341, *p* = 0.734). Participants in the NOPAIN group were older, had a higher socioeconomic status and had received the first diagnosis of EM a longer time ago than participants in the CPP group. Participants with CPP were more likely to additionally suffer from other kinds of persistent pain. They also subjectively tolerated the hormonal treatment worse (more side effects) and reported less pain reduction from it.

Participants in the CPP group were more likely to suffer from each pelvic pain type. Further, they reported the intensity of each pelvic pain type to be higher and the need for pain relief to be greater. They were more likely to report their relationship to be negatively affected by the EM.

### 3.2. Mental Disorders, Sexual Dysfunctions and Childhood Maltreatment

**Figure 1:**
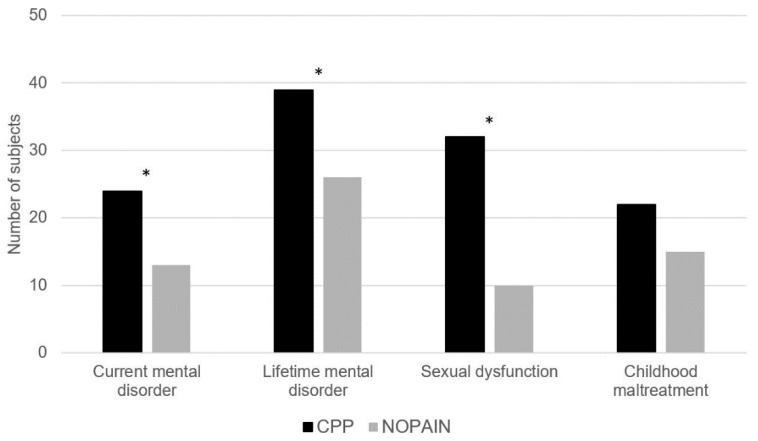
Rates of a current mental disorder (*p* = 0.019, odds ratio OR = 2.63), a lifetime mental disorder (*p* = 0.006, OR = 3.27), current sexual dysfunction (*p* < 0.001, OR = 7.11) and childhood maltreatment (CM) without the subscales physical neglect and trivialization (*p* = 0.074, OR = 2.04) in the groups CPP and NOPAIN. * *p* < 0.05.

The results are shown in Figure 1 and Table 2.

Participants with CPP were 2.63-times more likely to suffer from any current comorbid mental disorder and 7.98-times more likely to suffer from a somatic symptom disorder specifically. They were 2.46-times more likely to meet the diagnostic criteria of any former mental disorder and 3.02-times more likely to meet those of a former depressive disorder specifically. Regarding lifetime diagnoses of mental disorders, participants with CPP were 3.27-times more likely to receive any, 2.53-times to meet the criteria of an anxiety disorder, 4.15-times to meet the criteria of a depressive disorder and 7.98-times to receive a lifetime diagnosis of a somatic symptom disorder. Of the participants who met the criteria of a lifetime specific phobia (CPP: *n* = 10/50, 20%, NOPAIN: *n* = 2/50, 4%), 4/12 (33%; CPP: *n* = 2/10, 20%, NOPAIN: *n* = 2/2, 100%) presented with phobias related to gynecological examinations or treatments (e.g., surgeries). Of the women who reported current (CPP: *n* = 1/50, 2%, NOPAIN: *n* = 1/50, 2%) or former suicidal thoughts (CPP: *n* = 21/50, 42%, NOPAIN: *n* = 10/50, 20%), 10/31 (32%; CPP: *n* = 7/21, 33%, NOPAIN: *n* = 3/10, 30%) specified EM or related pain as the trigger.

Participants with CPP were 7.11-times more likely to report any sexual dysfunction. They were also more likely to suffer from each sexual dysfunction specifically, with odds ratios between 4.33 and 19.06.

No difference between the groups was found regarding the frequency of CM in general (*p* = 0.074); however, participants with CPP were 2.04-times more likely to report CM than participants in the NOPAIN group. Group comparisons did not meet statistical significance regarding the frequency of any kind of CM specifically (*p* = 0.095–0.500) or the number of CM rates (*p* = 0.547). The two groups did not significantly differ in the total sum score of the CTQ (*p* = 0.489) or the scores of any abuse or neglect subscale specifically (*p* = 0.117–0.890).

### 3.3. Prediction Models of a Current Mental Disorder

**Figure 2:**
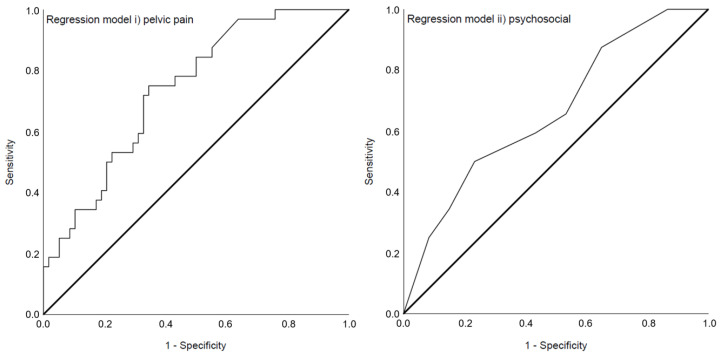
Receiver operating characteristics curve (ROC curve) showing the ability to predict a current mental disorder of the models i) pelvic pain with an area under the curve of 0.737 (95%-CI 0.635–0.840) and ii) psychosocial with an area under the curve of 0.663 (95%-CI 0.548–0.778).

Both regression analyses resulted in significant overall models (Table 3). The variance in the dependent variable current mental disorder explained by model i) pelvic pain (χ^2^ (3) = 14.27, *p* = 0.003) was 20.1% (Nagelkerke’s R^2^ = 0.201). The principal component resembling the need for pain relief (aOR = 4.08, *p* = 0.026) was a significant predictor in model i) and the area under the ROC curve (Figure 2) was 0.737 (95%-CI 0.635–0.840). Model ii) psychosocial (χ^2^ (3) = 7.89, *p* = 0.048) contributed to 11.3% (Nagelkerke’s R^2^ = 0.113) of the variance explanation of the dependent variable current mental disorder. Any current sexual dysfunction (aOR = 2.69, *p* = 0.031) was a significant predictor in this model and the area under the ROC curve (Figure 2) was 0.663 (95%-CI 0.548–0.778). Assessment of the assumption of no multicollinearity for both models is shown in Appendix B.

## 4. Discussion

In this study, women with EM and CPP were more likely to suffer from a current mental disorder and to report sexual dysfunction, but not CM, than women with EM and minimal to no pelvic pain (Table 2). This result confirms that CPP is not only a risk factor for psychopathological symptoms [10,11] but also for manifest mental disorders in women with EM. Both models to predict a current mental disorder set up in this study delivered significant results, highlighting the relevance of both pain-focused and psychosocial treatment in EM. In the presented analyses, a current mental disorder was associated with the extent of the need for pain relief and a current sexual dysfunction. These results demonstrate the importance of both pain reduction and the treatment of sexual impairment to maintain mental health in EM.

Women with CPP were more likely to meet the diagnostic criteria of both current and lifetime mental disorders. Seven (14%) women with CPP vs. one (2%) without pelvic pain met the criteria of a current somatic symptom disorder. Criteria of somatic symptom disorder (DSM-5) are (A) at least one somatic symptom that is distressing or results in significant disruption to daily life, (B) either 1. disproportionate and persistent thoughts about the seriousness of one’s symptoms, 2. a persistently high level of anxiety about health or symptoms or 3. excessive time and energy devoted to these symptoms and (C) criteria (A) and (B) persisting for at least 6 months. The majority of the participants did not display excessive thoughts, feelings or behaviors related to their somatic symptoms (CPP) and did not meet criterion B. On the contrary, women in our study presented with other mental disorders more frequently, as anxiety disorders were the most prevalent current mental disorders. These results are in line with former findings of women with EM being concerned with feelings of loss of control, powerlessness, uncertainty and disruption [29,30,31]. They underline the importance of establishing psychological counseling, addressing specific EM-related topics in treatment centers. The gap between current and lifetime depressive disorders may be explained by the recruitment procedure of this study, as people suffering from acute depression are less ready to actively respond to public study advertisements. The frequency of lifetime depressive disorders, however, corresponds to the results of high depressive symptoms from former studies [3,11]. Another interesting outcome is that no participant met the criteria for bipolar disorder. Further studies are necessary to clarify the possible association between EM and bipolar disorders, which has been discussed in previous research [5,6,7]. The number of participants who met the criteria of a lifetime specific phobia related to gynecological procedures and who specified EM or related pain as the trigger for suicidal thoughts highlights the urgency of pain relief as well as the need for gentleness in gynecological examinations. Clinicians should be aware of the possibility of patients’ desperation and not shy away from asking about suicidal thoughts and a referral to a mental health professional.

As almost half of the women with CPP compared to a third of those without pelvic pain reported CM, the possibility remains that a significant effect would be detected in a bigger sample, as suggested by former research on the association of CPP and CM [32]. However, the effect size of odds ratio = 2.04 for this association in our sample was lower than the effect sizes reported in this study of odds ratio = 3.2–4.3 [32]. Another possibility is that an association exists between CM and EM, which we did not examine, as no healthy control group was included in the study design. The rates of CM in our total sample, however, were lower than those in the EM groups reported in other studies [17,18]. In these studies, the effects of the association between EM and CM were odds ratios = 1.1–1.2 [18] and rate ratios = 1.20 and 1.49 [17]. As pointed out by Wischmann [33], patients affected by a disease without a clear pathogenesis, such as EM, strive for a sense of coherence and a regain of interpretative control, which can lead to individuals using any subjectively plausible explanation for their disease; therefore, the possibility of recall bias must be considered in the retrospective assessment of CM in EM patients. To exemplify this, in a recent study [34], when asked for their subjective theories of disease pathogenesis, the majority of women reported psychological factors, such as stress or worry, as a possible cause for their EM.

Women with CPP were, on average, 4 years younger, which could be the reason for the lower socioeconomic status and the shorter time since having received the first diagnosis of EM in this group (Table 1). Groups did not differ in the number of women reporting infertility and additional medical conditions, which could also be explained by the age difference, as fewer women try to conceive or are diagnosed with multiple medical conditions at a younger age. The age difference, however, is not explanatory of the fact that there was no difference in the percentage of women using hormonal treatment or specific hormonal substances. This result highlights the difficulty to find a hormonal substance with sufficient subjective tolerance and pain reduction for each patient. The resistance to hormonal treatment in the CPP group’s pain symptoms exemplifies the seriousness of central pain sensitization [35].

Due to the elaborate interview instrument used to assess mental disorders and sexual dysfunctions, our sample comprised 100 participants, a number comparable to similar studies [8,14]. However, the sample size in general and, more particularly, the number of women who met the criteria for a current mental disorder, limited the number of independent variables we could include in our regression models. To develop a more comprehensive prediction model of mental disorders in EM, including a larger number of independent variables, a bigger sample size would be required. Furthermore, from a statistical point of view, it has to be noted that the independent variables included in model i) pelvic pain (Table 3) showed high intercorrelations (Appendix B, Table A1). Highly intercorrelated predictor variables (multicollinearity) limit the statistical power of regression models as their independent contribution to the model is limited and should, therefore, be avoided. One possibility to deal with this is to exclude one or more of the predictor variables from the regression model; however, this should only be done according to theoretical considerations, as all intercorrelated variables are of equal statistical value. Therefore, there is no statistically accurate solution for which of the collinear variables to omit [27]. In the specific case of model i), despite the high intercorrelation, multicollinearity diagnostics resulted in yet acceptable values (Appendix B, Table A2). Further, the research question in this study was not exploratory but to test the two pre-defined models. Based on these considerations, no variables were omitted from our models. When interpreting model i), however, the high intercorrelations between the predictor variables should be kept in mind. Nevertheless, our models i) and ii) both met statistical significance and contributed to 20.1% (model i) pelvic pain) and 11.3% (model ii) psychosocial) of the explained variance in the dependent variable current mental disorder. Thus, our results demonstrate that both pelvic-pain-focused and psychosocial interventions are warranted in EM care. Future studies should take an integrative, biopsychosocial approach by building upon this result and further including biomarkers for which there are recent promising findings within research [36,37].

When examining the relationship between EM-related chronic pain and psychological distress, typically, the question arises of what came first. To answer this in a methodologically accurate way, however, large prospective cohort studies would be necessary where participants would be screened for EM, CPP and psychological distress from a young age on. In the direct query of the chronological development retrospectively, recall bias would pose a problem. Following Laganà et al. [38], rather than a unidirectional causation, a bidirectional relationship between mental health deterioration and CPP can be assumed in the form of a vicious circle (pelvic pain → worsening of mental health → worsening of pelvic pain → worsening of mental health). Finally, the research question addressed in this study is more concerned with women’s current situations to identify the need for support measures. Our results justify the demand for additional psychological support for women with EM in general and women with EM and CPP specifically. Still, our findings are preliminary and further studies are warranted to confirm them. Only women with either CPP or no to minimal pelvic pain were included in this study and self-selection bias may have led to an overrepresentation of women with mental disorders. Therefore, the results of this study can only be generalized to a limited extent. Further, recall bias in the assessment of CM is a possibility. These general limitations, however, do not diminish our findings, as our hypotheses were comparative and our further analyses were based on associations. Data on diagnostic delay [29] and individual differences, including self-esteem or self-efficacy [39], which have been shown to be associated with psychological distress in women with EM, were not included in this study. These omissions underline the complexity of the disease.

In conclusion, our results showed that CPP was associated with sexual dysfunctions and manifest mental disorders in women with EM. They highlight the need for pain relief and the establishment of counseling and patient training, with a particular focus on sexual impairment in EM care. Interdisciplinary care and cooperation between different medical and healthcare professions are both necessary to adequately treat this complex disease and to prevent mental health decompensation.

## Figures and Tables

**Table 1:** Sociodemographic, clinical, endometriosis-related and psychosocial characteristics in the groups CPP and NOPAIN.

		*Group*			
	*N*	CPP (*n* = 50) ^1^	NOPAIN (*n* = 50) ^1^	*p*-Value	Effect Size ^1^
***Sociodemographic data***					
Age	99	28.79 (5.59)	32.71 (6.26)	0.001 ^2,7^	d = 0.66
Socioeconomic status	90	10.65 (8.81; 12.04)	11.80 (10.60; 13.50)	0.008 ^3,7^	d = 0.57
Relationship	100	36 (72%)	43 (86%)	0.140 ^4,7^	
Children	100	7 (14%)	12 (24%)	0.154 ^4,6^	
University degree	98	22 (45%)	24 (49%)	0.840 ^4,7^	
***Clinical characteristics***					
Time since first diagnosis (years)	99	2.07 (1.34; 4.65)	3.76 (2.00; 7.21)	0.008 ^3,7^	d = 0.56
Number of surgeries	99	2.00 (1.00; 3.00)	1.00 (1.00; 2.00)	0.051 ^3,7^	
Hormonal treatment	100	34 (68%)	30 (60%)	0.532 ^4,7^	
Hormones: Mode of intake ^8^	63	-	-	0.525 ^4,7^	
Hormones: Substance ^9^	100	-	-	0.708 ^5,7^	
Hormones: Tolerance	54	2.00 (2.00; 3.00)	3.00 (3.00; 3.00)	<0.001 ^3,7^	d = 0.96
Hormones: Pain reduction	52	2.00 (1.50; 3.00)	5.00 (4.00; 5.00)	<0.001 ^3,6^	d = 1.85
Infertility	100	18 (36%)	15 (30%)	0.335 ^4,6^	
Other medical condition	100	27 (54%)	21 (42%)	0.158 ^4,6^	
Additional persistent pain	100	43 (86%)	14 (28%)	<0.001 ^4,6^	OR = 15.80
***Endometriosis***					
Pelvic pain days per month	100	30.50 (25.00; 31.00)	1.00 (0.00; 2.50)	<0.001 ^3,7^	d = 3.40
**Dysmenorrhea**	99	32 (65%)	17 (34%)	0.002 ^4,6^	OR = 3.65
Primary dysmenorrhea	38	29 (88%)	12 (80%)	0.378 ^5,6^	
Pain intensity	98	6.00 (0.00; 8.00)	0.00 (0.00; 1.00)	<0.001 ^3,7^	d = 1.05
Pain intensity: treatment-goal	98	3.00 (0.00; 4.00)	0.00 (0.00; 1.25)	<0.001 ^3,7^	d = 0.83
Pain intensity: discrepancy	97	2.00 (0.00; 4.00)	0.00 (0.00; 0.00)	<0.001 ^3,7^	d = 1.40
**Non-menstrual cyclical pelvic pain**	98	30 (63%)	10 (20%)	<0.001 ^4,6^	OR = 6.67
Duration (years)	36	10.00 (5.00; 15.00)	7.00 (4.75; 11.00)	0.351 ^3,7^	
Pain intensity	99	4.00 (0.00; 6.00)	0.00 (0.00; 0.00)	<0.001 ^3,7^	d = 1.08
Pain intensity: treatment-goal	99	2.00 (0.00; 3.00)	0.00 (0.00; 0.00)	<0.001 ^3,7^	d = 0.85
Pain intensity: discrepancy	98	2.00 (0.00; 3.00)	0.00 (0.00; 0.00)	<0.001 ^3,7^	d = 1.22
**Non-cyclical pelvic pain**	100	50 (100%)	11 (22%)	<0.001 ^4,6^	
Duration (years)	48	6.00 (3.00; 10.25)	5.00 (0.85; 7.25)	0.173 ^3,7^	
Pain intensity	97	5.00 (4.00; 6.00)	0.00 (0.00; 0.00)	<0.001 ^3,7^	d = 3.11
Pain intensity: treatment-goal	97	2.00 (1.00; 3.00)	0.00 (0.00; 0.00)	<0.001 ^3,7^	d = 1.49
Pain intensity: discrepancy	96	3.00 (2.00; 3.50)	0.00 (0.00; 0.00)	<0.001 ^3,7^	d = 2.88
Duration of CPP (years)	42	2.25 (1.50; 5.00)	-	-	
**Painful sexual intercourse**	100	46 (92%)	27 (54%)	<0.001 ^4,7^	OR = 9.80
Pain intensity	99	5.00 (3.00; 7.00)	0.00 (0.00; 2.00)	<0.001 ^3,7^	d = 1.82
Pain intensity: treatment-goal	99	1.50 (1.00; 3.00)	0.00 (0.00; 1.00)	<0.001 ^3,7^	d = 0.83
Pain intensity: discrepancy	99	3.00 (2.00; 4.00)	0.00 (0.00; 0.13)	<0.001 ^3,7^	d = 1.73
**Dyschezia**	100	40 (80%)	23 (46%)	<0.001 ^4,7^	OR = 4.70
Pain intensity	99	4.00 (2.00; 5.00)	0.00 (0.00; 2.00)	<0.001 ^3,7^	d = 1.15
Pain intensity: treatment-goal	98	2.00 (0.00; 2.00)	0.00 (0.00; 1.25)	<0.001 ^3,7^	d = 0.76
Pain intensity: discrepancy	98	2.00 (0.00; 3.00)	0.00 (0.00; 1.00)	<0.001 ^3,7^	d = 0.98
**Dysuria**	100	26 (52%)	7 (14%)	<0.001 ^4,7^	OR = 6.65
Pain intensity	100	0.75 (0.00; 4.00)	0.00 (0.00; 0.00)	<0.001 ^3,7^	d = 0.72
Pain intensity treatment-goal	99	0.00 (0.00; 2.00)	0.00 (0.00; 0.00)	<0.001 ^3,7^	d = 0.66
Pain intensity: discrepancy	99	0.00 (0.00; 2.00)	0.00 (0.00; 0.00)	<0.001 ^3,7^	d = 0.58
***Psychosocial characteristics***					
Affected career	98	24 (49%)	16 (33%)	0.075 ^4,7^	
Affected relationship	95	42 (89%)	24 (50%)	<0.001 ^4,7^	OR = 8.4

^1^ Data presented as M (SD), Median (percentile 25; percentile 75) or frequencies (%), d = Cohen’s d, OR = odds ratio. ^2^ t-test, ^3^ Mann–Whitney U-test, ^4^ χ^2^-test, ^5^ Fisher’s exact test, ^6^ one-tailed testing, ^7^ two-tailed testing. ^8^ mode of intake: non-stop: CPP = 28 (85%), NOPAIN = 23 (77%); cyclical: CPP = 5 (15%), NOPAIN = 7 (23%). ^9^ substance: no hormonal treatment: CPP = 17 (34%), NOPAIN = 20 (40%); COC: CPP = 9 (18%), NOPAIN = 10 (20%); desogestrel: CPP = 3 (6%), NOPAIN = 6 (12%); dienogest: CPP = 15 (30%), NOPAIN = 11 (22%); GnRHA: CPP = 3 (6%), NOPAIN = 1 (2%); hormone-releasing IUD: CPP = 3 (6%), NOPAIN = 2 (4%).

**Table 2:** Testing of hypotheses: comparisons of the rates of mental disorders, sexual dysfunctions and childhood maltreatment in the groups CPP and NOPAIN.

		*Group*			
	*N*	CPP (*n* = 50) ^1^	NOPAIN (*n* = 50) ^1^	*p*-Value	Effect Size ^1^
**Current mental disorder**	100	24 (48%)	13 (26%)	0.019 ^2,4^	2.63
Anxiety	100	19 (38%)	11 (22%)	0.063 ^2,4^	
Bipolar	100	-	-	-	
Depressive	100	5 (10%)	2 (4%)	0.218 ^3,4^	
Obsessive-compulsive and related	100	1 (2%)	2 (4%)	0.500 ^3,4^	
Trauma- and stressor related	100	4 (8%)	1 (2%)	0.181 ^3,4^	
Somatic symptom and related	100	7 (14%)	1 (2%)	0.030 ^3,4^	7.98
Feeding and eating	100	1 (2%)	-	0.500 ^3,4^	
Substance-related and addictive	100	-	-	-	
**Former mental disorder**	100	32 (64%)	21 (42%)	0.022 ^2,4^	2.46
Anxiety	100	7 (14%)	7 (14%)	0.613 ^2,4^	
Bipolar	100	-	-	-	
Depressive	100	27 (54%)	14 (28%)	0.007 ^2,4^	3.02
Obsessive-compulsive and related	100	-	1 (2%)	0.500 ^3,4^	
Trauma- and stressor related	100	-	-	-	
Somatic symptom and related	100	-	-	-	
Feeding and eating	100	5 (10%)	1 (2%)	0.102 ^3,4^	
Substance-related and addictive	100	1 (2%)	1 (2%)	0.753 ^3,4^	
**Lifetime mental disorder**	100	39 (78%)	26 (52%)	0.006 ^2,4^	3.27
Anxiety	100	26 (52%)	15 (30%)	0.021 ^2,4^	2.53
Bipolar	100	-	-	-	
Depressive	100	32 (64%)	15 (30%)	0.001 ^2,4^	4.15
Obsessive-compulsive and related	100	1 (2%)	3 (6%)	0.309 ^3,4^	
Trauma- and stressor related	100	4 (8%)	1 (2%)	0.181 ^3,4^	
Somatic symptom and related	100	7 (14%)	1 (2%)	0.030 ^3,4^	7.98
Feeding and eating	100	6 (12%)	1 (2%)	0.056 ^3,4^	
Substance-related and addictive	100	1 (2%)	1 (2%)	0.753 ^3,4^	
**Sexual dysfunction**	100	32 (64%)	10 (20%)	<0.001 ^2,4^	7.11
Sexual Desire	100	26 (52%)	10 (20%)	0.001 ^2,4^	4.33
Sexual Arousal	100	24 (48%)	7 (14%)	<0.001 ^2,4^	5.67
Orgasmic	100	20 (40%)	6 (12%)	0.001 ^2,4^	4.89
Dyspareunia	100	32 (64%)	8 (16%)	<0.001 ^2,4^	9.33
Vaginismus	100	14 (28%)	1 (2%)	<0.001 ^2,4^	19.06
**Rates: childhood maltreatment CM ^7^**	92	22 (49%)	15 (32%)	0.074 ^2,4^	2.04
Emotional abuse	96	12 (25%)	6 (12%)	0.095 ^2,4^	
Physical abuse	98	5 (10%)	4 (8%)	0.500 ^3,4^	
Sexual abuse	91	9 (21%)	5 (11%)	0.157 ^2,4^	
Emotional neglect	96	6 (13%)	8 (16%)	0.420 ^2,4^	
Physical neglect	95	23 (49%)	26 (54%)	0.380 ^2,4^	
**Scales: CTQ ^8^**	90	29.00 (24.00; 35.00)	28.00 (23.00; 33.00)	0.437 ^5,6^	
Emotional abuse	96	8.50 (7.00; 12.75)	9.00 (6.25; 10.00)	0.472 ^5,6^	
Physical abuse	98	4.00 (4.00; 4.00)	4.00 (4.00; 4.00)	0.890 ^5,6^	
Sexual abuse	91	5.00 (5.00; 7.00)	5.00 (5.00; 5.00)	0.117 ^5,6^	
Emotional neglect	96	9.00 (7.00; 12.00)	9.00 (6.00; 12.00)	0.822 ^5,6^	
Physical neglect	95	9.00 (9.00; 11.00)	10.00 (9.00; 11.00)	0.554 ^5,6^	
Trivialization	95	0.00 (0.00; 0.00)	0.00 (0.00; 0.75)	0.380 ^5,6^	
Number of CM ^7^	92	1.00 (0.00; 2.00)	1.00 (0.00; 2.00)	0.547 ^5,6^	

^1^ Data presented as Median (percentile 25; percentile 75) or frequencies (%), effect size = odds ratio. ^2^ χ^2^-test, ^3^ Fisher’s exact test, ^4^ one-tailed testing, ^5^ Mann–Whitney U-test, ^6^ two-tailed testing. ^7^ Childhood maltreatment CM = any CM without the subscales physical neglect and trivialization. ^8^ CTQ = Childhood Trauma Questionnaire, total sum score without the subscale physical neglect.

**Table 3:** Results of the binary-logistic regression analyses for the cross-sectional prediction of a current comorbid mental disorder in the total sample including (i) pelvic-pain-related predictor variables or (ii) psychosocial predictor variables.

Variable	B (Std.-Error) ^1^	*p*-Value	aOR ^1^	95%-CI ^1^
***Model i) pelvic pain* ^2^**				
Pelvic pain days per month	−0.01 (0.04)	0.737	0.99	0.92–1.06
PC_intensity ^3^	−0.43 (0.67)	0.522	0.65	0.18–2.42
PC_painrelief ^4^	1.41 (0.63)	0.026	4.08	1.19–14.04
Constant	−0.49 (0.57)	0.394	0.61	
***Model ii) psychosocial* ^5^**				
Childhood maltreatment CM ^6^	−0.14 (0.47)	0.768	0.87	0.35–2.19
Former mental disorder	0.84 (0.47)	0.073	2.31	0.92–5.78
Any sexual dysfunction	0.99 (0.46)	0.031	2.69	1.09–6.64
Constant	−1.49 (0.47)	0.001	0.23	

^1^ B = unstandardized beta coefficient, Std. Error = standard error of the unstandardized beta coefficient, aOR = adjusted odds ratio, 95%-CI = 95% confidence interval. ^2^ *n* = 90, Nagelkerke’s R^2^ = 0.201; model i: χ^2^ (3) = 14.27, *p* = 0.003; Hosmer–Lemeshow goodness-of-fit test: χ^2^ (8) = 8.69, *p* = 0.369. ^3^ PC_intensity = principal component for the pelvic pain intensity. ^4^ PC_painrelief = principal component resembling the need for pain relief. ^5^ *n* = 92, Nagelkerke’s R^2^ = 0.113; model ii: χ^2^ (3) = 7.89, *p* = 0.048; Hosmer–Lemeshow goodness-of-fit test: χ^2^ (6) = 8.10, *p* = 0.231. ^6^ Childhood maltreatment CM = any CM without the subscales physical neglect and trivialization.

## Data Availability

We do not have the ethics committee’s or our participants’ consent to make the data acquired and analyzed in this study publicly available. The data are available from the corresponding author on reasonable request.

## Appendix A

For pain intensity, the Kaiser–Meyer–Olkin measure verified the sampling adequacy for the analysis, KMO = 0.78, and all KMO values for individual pain intensity scores were >0.70 and, thus, above the acceptable limit of 0.5. Bartlett’s test of sphericity χ^2^ (15) = 211.64, *p* < 0.001, indicated that correlations between the individual pain scores were large enough for PCA. The component for pain intensity (new variable PC_intensity) explained 53.62% of the variance with loadings of 0.52–0.78. For pain relief, the Kaiser–Meyer–Olkin measure verified the sampling adequacy for the analysis, KMO = 0.76, and all KMO values for individual discrepancy scores were >0.69 and, thus, above the acceptable limit of 0.5. Bartlett’s test of sphericity χ^2^ (15) = 201.82, *p* < 0.001, indicated that correlations between the individual discrepancy scores were large enough for PCA. The component describing the need for pain relief (new variable PC_painrelief) explained 51.77% of the variance with loadings of 0.44–0.82.

## Appendix B

To test for multicollinearity in the independent variables in regression models i) pelvic pain and ii) psychosocial, correlation analyses between the predictor variables (Table A1), the variance inflation factors (VIF) and tolerance statistics (Table A2) are presented. According to Field [27], VIF values >10 and tolerance values < 0.1 indicate serious collinearity problems; if the average VIF value is substantially >1, the regression may be biased and tolerance values < 0.2 indicate potential collinearity problems.

**Table A1:** Nonparametric bivariate correlation analyses between the independent predictor variables in the models i) pelvic pain and ii) psychosocial.

Variable	Variable		
***Model i) pelvic pain***	Pelvic pain days per month	PC_intensity ^1^	PC_painrelief ^2^
Pelvic pain days per month	1.00	0.864 **	0.762 **
PC_intensity ^1^	0.864 **	1.00	0.900 **
PC_painrelief ^2^	0.762 **	0.900 **	1.00
***Model ii) psychosocial***	Childhood maltreatment CM ^3^	Former mental disorder	Any sexual dysfunction
Childhood maltreatment CM ^3^	1.00	0.057	−0.013
Former mental disorder	0.057	1.00	0.030
Any sexual dysfunction	−0.013	0.030	1.00

^1^ PC_intensity = principal component for the pelvic pain intensity. ^2^ PC_painrelief = principal component resembling the need for pain relief. ^3^ Childhood maltreatment CM = any CM without the subscales physical neglect and trivialization. ** *p* < 0.01.

**Table A2:** Results of collinearity diagnostics for models i) pelvic pain and ii) psychosocial.

	Collinearity Statistics	
Variable	Tolerance	VIF
***Model i) pelvic pain***		
Pelvic pain days per month	0.247	4.048
PC_intensity ^1^	0.134	7.457
PC_painrelief ^2^	0.194	5.148
***Model ii) psychosocial***		
Childhood maltreatment CM ^3^	0.997	1.003
Former mental disorder	0.997	1.003
Any sexual dysfunction	1.00	1.00

^1^ PC_intensity = principal component for the pelvic pain intensity. ^2^ PC_painrelief = principal component resembling the need for pain relief. ^3^ Childhood maltreatment CM = any CM without the subscales physical neglect and trivialization.

## References

[1] Bulun S.E. (2018). Endometriosis. Yen & Jaffe’s Reproductive Endocrinology: Physiology, Pathophysiology, and Clinical Management.

[2] Mechsner S. (2016). Endometriose. Schmerz.

[3] Laganà A.S., Condemi I., Retto G., Muscatello M.R.A., Bruno A., Zoccali R.A., Triolo O., Cedro C. (2015). Analysis of psychopathological comorbidity behind the common symptoms and signs of endometriosis. Eur. J. Obstet. Gynecol. Reprod. Biol..

[4] Sepulcri R.d.P., do Amaral V.F. (2009). Depressive symptoms, anxiety, and quality of life in women with pelvic endometriosis. Eur. J. Obstet. Gynecol. Reprod. Biol..

[5] Lewis D.O., Comite F., Mallouh C., Zadunaisky L., Hutchinson-Williams K., Cherksey B.D., Yeager C. (1987). Bipolar mood disorder and endometriosis: Preliminary findings. Am. J. Psychiatry.

[6] Walker E., Katon W., Jones L.M., Russo J. (1989). Relationship between endometriosis and affective disorder. Am. J. Psychiatry.

[7] Kumar V., Khan M., Vilos G.A., Sharma V. (2011). Revisiting the association between endometriosis and bipolar disorder. J. Obs. Gynaecol. Can..

[8] Cavaggioni G., Lia C., Resta S., Antonielli T., Panici P.B., Megiorni F., Porpora M.G. (2014). Are mood and anxiety disorders and alexithymia associated with endometriosis? A preliminary study. Biomed. Res. Int..

[9] Rief W., Stenzel N., Berking M., Rief W. (2012). Diagnostik Und Klassifikation. Klinische Psychologie und Psychotherapie für Bachelor—Band I: Grundlagen und Störungswissen.

[10] Gambadauro P., Carli V., Hadlaczky G. (2019). Depressive symptoms among women with endometriosis: A systematic review and meta-analysis. Am. J. Obstet. Gynecol..

[11] van Barneveld E., Manders J., van Osch F., van Poll M., Visser L., Hanegem L., Lim A., Bongers M., Leue C. (2021). Depression, anxiety and correlating factors in endometriosis: A systematic review and meta-analysis. J. Womens Health.

[12] de Graaff A.A., van Lankveld J., Smits L.J., van Beek J.J., Dunselman G.A.J. (2016). Dyspareunia and depressive symptoms are associated with impaired sexual functioning in women with endometriosis, whereas sexual functioning in their male partners is not affected. Hum. Reprod..

[13] Jia S., Leng J., Sun P., Lang J. (2013). Prevalence and associated factors of female sexual dysfunction in women with endometriosis. Obstet. Gynecol..

[14] Osório F.L., Carvalho A.C.F., Donadon M.F., Moreno A.L., Polli-Neto O. (2016). Chronic pelvic pain, psychiatric disorders and early emotional traumas: Results of a cross sectional case-control study. World J. Psychiatry.

[15] Latthe P., Mignini L., Gray R., Hills R., Khan K. (2006). Factors predisposing women to chronic pelvic pain: Systematic review. BMJ.

[16] McCrory E.J., Viding E. (2015). The theory of latent vulnerability: Reconceptualizing the link between childhood maltreatment and psychiatric disorder. Dev. Psychopathol..

[17] Harris H.R., Wieser F., Vitonis A.F., Rich-Edwards J., Boynton-Jarrett R., Bertone-Johnson E.R., Missmer S.A. (2018). Early life abuse and risk of endometriosis. Hum. Reprod..

[18] Liebermann C., Schwartz A.S.K., Charpidou T., Geraedts K., Rauchfuss M., Wöl M., Von Orelli S., Häberlin F., Eberhard M., Imesch P. (2018). Maltreatment during childhood: A risk factor for the development of endometriosis?. Hum. Reprod..

[19] Schliep K.C., Mumford S.L., Johnstone E.B., Peterson C.M., Sharp H.T., Stanford J.B., Chen Z., Backonja U., Wallace M.E., Buck Louis G.M. (2016). Sexual and physical abuse and gynecologic disorders. Hum. Reprod..

[20] Haas D., Shebl O., Shamiyeh A., Oppelt P. (2013). The rASRM score and the ENZIAN classification for endometriosis: Their strengths and weaknesses. Acta Obstet. Gynecol. Scand..

[21] Margraf J., Cwik J.C., Suppiger A., Schneider S. (2017). DIPS Open Access: Diagnostic Interview for Mental Disorders.

[22] American Psychiatric Association (2013). Diagnostic and Statistical Manual of Mental Disorders.

[23] Margraf J., Cwik J.C., Pflug V., Schneider S. (2017). Structured clinical interviews for mental disorders across the lifespan: Psychometric quality and further developments of the DIPS Open Access Interviews. Z. Klin. Psychol. Psychother..

[24] Lampert T., Kroll L.E., Müters S., Stolzenberg H. (2013). Messung des sozioökonomischen Status in der Studie “Gesundheit in Deutschland Aktuell“ (GEDA). Bundesgesundheitsblatt.

[25] Klinitzke G., Romppel M., Häuser W., Brähler E., Glaesmer H. (2012). The German version of the Childhood Trauma Questionnaire (CTQ)—Psychometric characteristics in a representative sample of the general population. Psychother. Psychosom. Med. Psychol..

[26] IBM Corp (2019). IBM SPSS Statistics for Windows, Version 26.0.

[27] Field A. (2009). Discovering Statistics Using SPSS.

[28] Márki G., Bokor A., Rigó J., Rigó A. (2017). Physical pain and emotion regulation as the main predictive factors of health-related quality of life in women living with endometriosis. Hum. Reprod..

[29] Facchin F., Saita E., Barbara G., Dridi D., Vercellini P. (2017). “Free butterflies will come out of these deep wounds”: A grounded theory of how endometriosis affects women’s psychological health. J. Health Psychol..

[30] Denny E. (2009). “I never know from one day to another how I will feel”: Pain and uncertainty in women with endometriosis. Qual. Health Res..

[31] Jones G., Jenkinson C., Kennedy S. (2004). The impact of endometriosis upon quality of life: A qualitative analysis. J. Psychosom. Obstet. Gynecol..

[32] Krantz T.E., Andrews N., Petersen T.R., Dunivan G.C., Montoya M., Swanson N., Wenzl C.K., Zambrano J.R., Komesu Y.M. (2019). Adverse childhood experiences among gynecology patients with chronic pelvic pain. Obstet. Gynecol..

[33] Wischmann T. (2008). Psychologische Aspekte bei Endometriose und Kinderwunsch—Einige kritische Anmerkungen. Geburtshilfe Frauenheilkd..

[34] Münch F., Ebert A.D., Mechsner S., Richter R., David M. (2022). Subjective theories of illness in fibroid and endometriosis patients: Similarities, differences, and influencing factors. J. Endometr. Pelvic Pain Disord..

[35] Hoffman D. (2015). Central and peripheral pain generators in women with chronic pelvic pain: Patient centered assessment and treatment. Curr. Rheumatol. Rev..

[36] Hortu I., Ozceltik G., Karadadas E., Erbas O., Yigitturk G., Ulukus M. (2020). The role of ankaferd blood stopper and oxytocin as potential therapeutic agents in endometriosis: A rat model. Curr. Med. Sci..

[37] Karadadas E., Hortu I., Ak H., Ergenoglu A.M., Karadadas N., Aydin H.H. (2020). Evaluation of complement systems proteins C3a, C5a and C6 in patients of endometriosis. Clin. Biochem..

[38] Laganà A.S., La Rosa V.L., Rapisarda A.M.C., Valenti G., Sapia F., Chiofalo B., Rossetti D., Frangez H.B., Bokal E.V., Vitale S.G. (2017). Anxiety and depression in patients with endometriosis: Impact and management challenges. Int. J. Womens Health.

[39] Facchin F., Barbara G., Dridi D., Alberico D., Buggio L., Somigliana E., Saita E., Vercellini P. (2017). Mental health in women with endometriosis: Searching for predictors of psychological distress. Hum. Reprod..

